# Dissecting Vegetative Period Into Its Phenotypic and Genotypic Components Allows Environment‐Specific Breeding in Lentil ( *Lens culinaris* Medik)

**DOI:** 10.1111/ppl.70729

**Published:** 2025-12-29

**Authors:** Salvador Osuna‐Caballero, Taryn Heidecker, James L. Weller, Kirstin E. Bett

**Affiliations:** ^1^ Department of Plant Sciences University of Saskatchewan Saskatoon Saskatchewan Canada; ^2^ School of Natural Sciences University of Tasmania Hobart Australia

**Keywords:** emergence, flowering, lentil breeding, QTL mapping, vegetative period

## Abstract

In legumes, flowering time is regulated by genes responsive to temperature and photoperiod, presenting challenges for high‐latitude lentil producers who must adapt cultivars to short growing seasons and extended daylight hours. Therefore, prolonged vegetative periods are favored in those areas. To address this, we studied a recombinant inbred line (RIL) population, derived from a cross between the adapted cultivar CDC‐Milestone and the non‐adapted line ILL8006, to investigate phenology‐related traits under long‐day conditions in western Canada. Significant variation in days to emergence (DTE), days to flowering (DTF), and days to pod maturity (DTM) enabled analysis of the vegetative (VegP) and reproductive (RepP) periods within the population. We constructed a high‐density genetic linkage map using molecular markers linked to genes in the Lcu.2RBY reference genome, identifying quantitative trait loci (QTLs) for those traits across four site‐years in Saskatchewan. Differential expression analysis of known flowering time genes enhanced interpretation of the QTL results for flowering time. Three major DTE QTLs (*qDTE2/3.II*, *qDTE2/3.III*, and *qDTE2/3.IV*) on chromosome 2 explained 16%–28% phenotypic variability, depending on the environment, with *in silico* analysis identifying six curated genes as putative candidates within that region. A key DTF QTL (*qDTF6.I*) on chromosome 6 accounted for 23%–56% of phenotypic variability, harboring a homolog of the *FLOWERING LOCUS T* gene, whose role was explored alongside other candidate genes. Dissecting the vegetative period into DTE and DTF revealed distinct genetic controls for each trait, enabling breeders to combine early or late emergence and flowering to optimize adaptation and yield in diverse agroclimatic conditions.

## 1 Introduction

Lentil ( *Lens culinaris* Medik. subsp. *culinaris*, 2*n* = 2*x* = 14) is an important crop in western Canada, where the annual production in Saskatchewan over the past decade has exceeded that of any other country (Wang 2024; FAOSTAT 2025). In this region, breeding programs have focused on enhancing agronomic and end‐use quality traits by using the existing pool of adapted genetic material (Tullu et al. 2013). The introgression of alleles from non‐adapted sources, that is, from wild lines or from material adapted to a different growing environment, could offer novel variation for improvement (Khazaei et al. 2016). However, Canadian breeders have avoided this approach due to the associated potential for introduction of other detrimental traits, particularly related to adaptation, which are difficult to overcome (Khazaei et al. 2016). A major challenge in incorporating global germplasm into a breeding program is the variation in phenology‐related traits (Erskine et al. 1989). Studies of diverse lentil accessions from worldwide origins demonstrate that genetic background significantly influences developmental responses to environmental cues (Wright et al. 2020), complicating the prediction of days to flowering (DTF) across genotypes in a single environment. However, integrating knowledge of major flowering time gene homologs identified in other crop species has been shown to improve these predictions (Bhakta et al. 2017; Cockram et al. 2007; Wenden et al. 2009).

Dissecting lentil growth and development into its distinct phases can highlight the potential for distinct genetic control of phenology‐related traits. These phases include the vegetative period (VegP) and the reproductive period (RepP), each of which includes distinct stages based on emergence, flowering, pod and seed development, and maturation time (Erskine et al. 1990). The VegP starts with seedling emergence, a growth phase influenced by seed dormancy and seed germination‐related traits. In annual crops, seed dormancy is considered a negative characteristic that breeders try to overcome, as it inhibits germination even under favorable conditions. By reducing the duration of dormancy, the days to emergence (DTE) trait is shortened, promoting a fast and uniform establishment of the crop. This uniformity has agronomic advantages like minimizing the competition with weeds. The DTE trait has often been excluded in quantitative genetic analysis due to the low variability usually observed in the field, especially relative to the larger differences in VegP driven by flowering time (Wright et al. 2020). Nevertheless, at least one study has reported QTL for DTE under controlled conditions (Rajandran et al. 2022), and it is a trait worth exploring further, as studies in different legumes have shown that faster emergence is positively correlated with higher yields and early flowering (De Ron et al. 2016; Oloyede‐Kamiyo et al. 2024). Physical dormancy in legumes is primarily caused by a water‐impermeable seed coat, especially the palisade (macrosclereid) layer, which is dense and thick in dormant seeds (Wen et al. 2024). Specifically in lentils, researchers have shown that DTE can be reduced through the exogenous application of gibberellins or abscisic acid antagonists, particularly in wild relatives where dormancy is more pronounced (Gorim et al. 2025). This response highlights the role of hormone‐mediated pathways in breaking dormancy and accelerating germination, revealing that genes involved in phytohormone biosynthesis, perception, and signaling could be implicated in regulating emergence time.

A significant limitation of previous genetic marker studies in lentils has been the anonymity of markers and their lack of transferability beyond the specific populations in which they were identified (Saha et al. 2013). Genetic maps that employ markers readily comparable to gene‐based maps are more likely to be applicable across diverse germplasm, thereby enhancing the reliability of marker‐assisted selection (MAS). This approach is increasingly being adopted in lentil research, with studies utilizing gene‐linked markers to target traits such as seed quality (Fedoruk et al. 2013; Jha et al. 2017; Verma et al. 2015) and micronutrient accumulation (Aldemir et al. 2017; Ates et al. 2016, 2018; Khazaei et al. 2017). The underutilization of genetic markers for phenology‐related trait screening in breeding programs can be attributed, in part, to the scarcity of mapping studies employing markers that are easily linked to gene‐based maps (Fedoruk et al. 2013; Haile et al. 2021; Kahriman et al. 2015). Gene‐linked markers also enable breeders to leverage insights from related species, taking advantage of the conserved nature of flowering genes across legumes (Weller et al. 2012). This conservation makes the extensive molecular flowering pathway knowledge from pea ( *Pisum sativum* ), barrel clover ( *Medicago truncatula* ), and, to a lesser extent, soybean ( *Glycine max* ), valuable resources for lentil improvement (Kim et al. 2013; Weller and Ortega 2015).

Among the well‐known flowering‐related genes in legumes are *FTa1*, *FTb2*, and *FTc*, which belong to the *FLOWERING LOCUS T* (*FT*) gene family and play critical roles in regulating floral induction. *FTa1* acts as a key integrator of photoperiod signals, promoting flowering under long‐day conditions in species like pea and barrel clover (Hecht et al. 2011; Laurie et al. 2011). *FTb2* is similarly involved in the transition to flowering under long‐day photoperiods, integrating inputs from the circadian clock and light quality (Thomson et al. 2019; Weller and Ortega 2015). *FTc*, though less extensively studied, appears to contribute to flowering time regulation, potentially by responding to temperature or other environmental cues (Ortega et al. 2019). These genes work together, with their expression patterns and interactions finely tuning the timing of flowering in response to environmental stimuli. Understanding their functions and interactions in lentils could provide essential insights into the genetic control of phenology‐related traits, specifically DTF, across diverse climatic conditions. Heritability estimates for DTF are typically moderate, reflecting its quantitative nature. Previous trials in Saskatchewan, based on narrow crosses, have reported DTF heritability estimates ranging from 30% to 53% (Tullu et al. 2008; Fedoruk et al. 2013; Haile et al. 2021).

This study used a Recombinant Inbred Line (RIL) population to identify genomic regions contributing to variation in various phenology‐related traits, including DTE, DTF, and days to pod maturity (DTM), under Saskatchewan field conditions (long days, moderate temperatures). This LR‐11 population originated from a broad cross between the South Asian‐developed ILL 8006 and the Canadian cultivar CDC Milestone. Incorporating multiple phenology‐related traits alongside DTF was expected to provide critical context for interpreting the QTLs and candidate genes identified in this study.

## 2 Materials and Methods

### 2.1 Plant Material

The *Lens culinaris* Medik. LR‐11 RIL population was developed at the Crop Development Centre (CDC), University of Saskatchewan, by crossing the South Asian line ILL 8006 with the temperate variety CDC Milestone and carrying individuals through single seed descent from F_2_ to F_8_ before bulking individuals to create RILs. The population available for use in this study consisted of 120 RILs.

ILL 8006, also called BariMasur‐4, is small‐seeded, with orange‐red cotyledons and a dotted gray seed coat (Kumar 2007; Sarker et al. 1999). It was developed in Bangladesh from a cross between ILL 5888 and ILL 5782 (Sarker et al. 1999). ILL 5888 was a selection from a Bangladeshi landrace and, interestingly, is one of the first lines to flower in a diversity panel in both South Asian and Saskatchewan trials (Wright et al. 2020). ILL 5888 is a source of resistance to Ascochyta blight, while ILL 5782 is a source for tolerance to rust and Stemphylium blight, both bred at ICARDA (Erskine et al. 1994; Sharpe et al. 2013). Neither ILL 5888 nor ILL 8006 is adapted to the northern temperate climate of Saskatchewan summers, and both suffer due to photoperiod insensitivity, meaning they flower exceedingly early under long days, an undesirable trait for western Canadian environments.

The Saskatchewan‐adapted parent, CDC Milestone, was developed by selecting a line from the cross of Eston × C8L27Y. C8L27Y was a breeding line used at Usask, which arose from a selection of the F_5_ generation in an F_2_‐derived population created by crossing Eston and ILL 5588. CDC Milestone is small‐seeded, with yellow cotyledons and a pale green seed coat with faint mottling (Vandenberg et al. 2001).

### 2.2 Phenotyping

This study was conducted at two sites in North‐Central Saskatchewan: Sutherland (GPS: 52.17, −106.51) and Rosthern (GPS: 52.68, −106.29). Climatic variables were downloaded from Historical Data—Climate—Environment and Climate Change Canada for the station named SASKATOON RCS at GPS coordinates: 52.17, −106.72. In 2017, the Sutherland site was seeded on May 4th and Rosthern was seeded on May 19th. In 2018, Sutherland was seeded May 9th and Rosthern was seeded May 11th.

The 120 RIL lines were sown in a randomized complete block design with three replications at each site. Each 1 m^2^ microplot was seeded with 120 seeds, provided there were enough seeds from previous harvests to allow it. In 2017, line LR‐11‐133 had only enough seeds for two replications (Sutherland Rep 1 and 2) and was seeded at 60 seeds per plot. In 2018, both parents were included in the trial design, but only CDC Milestone was included in 2017 due to a lack of ILL 8006 seeds.

The plots were visited every 1–3 days and phenotypes were called on the day 10% of the plot emerged (DTE), flowered (DTF), or matured (DTM). Vegetative Period (VegP) and Reproductive Period (RepP) were calculated by taking the difference between DTF and DTE, and DTM and DTF, respectively. Accumulated average daily temperature from sowing to DTE (DTE_T) was calculated (accumulated °C). Thermal growing time was also calculated for DTF and DTM as growing degree days (GDD_F and GDD_M, respectively). Total plot seed yield (YLD) was collected on lightly cleaned samples following harvest and threshing. Raw phenotypic data used in this study can be found at https://knowpulse.usask.ca/research‐study/Lens‐flowering‐LR11‐LDP.

### 2.3 Statistical Analyses

The years and locations of the field trials were combined and used as site‐years, hereinafter referred to as environments. All statistical analyses were done using the software R v4.3.3. (R Development Core Team 2018) with the ‘metan’ and ‘lme4’ R packages (Bates et al. 2015; Olivoto and Lúcio 2020). Variance components were calculated for each trait and environment using a one‐way linear mixed model treating the replications and the genotypes as random factors. This approach also facilitated the calculation of the predicted means for each genotype, termed best linear unbiased predictors (BLUPs), which were used in subsequent QTL analyses.

A second linear mixed model was applied using a multi‐environment trial (MET) framework. In this model, both the genotype and genotype–environment interaction (GEI) were treated as random effects, with all other sources of variance modelled as fixed effects. This model enabled the estimation of BLUPs for each trait across the four environments, representing the GEI‐independent component. Heritability (H^2^) in the MET model was calculated as the heritability on the mean basis, estimated by:

H2=σ^gen2σ^gen2+σ^int2e+σ^res2eb

where ${\hat{\sigma }}_{\mathrm{gen}}^{2}$ is the genotypic variance; ${\hat{\sigma }}_{\mathrm{int}}^{2}$ is the genotype‐by‐environment interaction variance; and ${\hat{\sigma }}_{\mathrm{res}}^{2}$ is the residual variance. *e* and *b* are the number of environments and replicates, respectively. The coefficient of determination of the genotype‐by‐environment effect, $r_{i}^{2}$, was calculated as:

$$r_{i}^{2}=\frac{{\hat{\sigma }}_{\mathrm{int}}^{2}}{{\hat{\sigma }}_{\mathrm{gen}}^{2}+{\hat{\sigma }}_{\mathrm{int}}^{2}+{\hat{\sigma }}_{\mathrm{res}}^{2}}$$

Finally, the genotype‐by‐environment correlation, $r_{\mathrm{ge}}^{2}$, was estimated by:

$$r_{\mathrm{ge}}^{2}=\frac{{\hat{\sigma }}_{\mathrm{gen}}^{2}}{{\hat{\sigma }}_{\mathrm{gen}}^{2}+{\hat{\sigma }}_{\mathrm{int}}^{2}}$$

### 2.4 LR‐11 Linkage Map Construction

All 120 lines and parents of the LR‐11 population were genotyped using a custom lentil exome capture assay (Ogutcen et al. 2018). Using an in‐house pipeline, reads were aligned to the CDC Redberry lentil genome v2.0 (Lcu.2RBY; Ramsay et al. 2021) to identify single nucleotide polymorphisms (SNPs), which were then made available for this study.

Exome capture‐SNP markers were filtered for those with < 10% missing or heterozygous calls. Afterward, only markers that were also present in the AGILE‐LDP genotyping results (Haile et al. 2020) were retained. This was done as an additional marker quality check and ensures identified markers were not variants unique to the bi‐parental population and, therefore, more useful for future studies. Markers mapping to unanchored contigs in the current assembly were included without the requirement of being found in the AGILE‐LDP, provided only one line at most scored heterozygous and < 10% of lines were missing an allele call.

SimpleMap (Jighly et al. 2015) was used to combine markers into bins of co‐segregating markers prior to mapping. SimpleMap retains the repulsion information within each bin, which allows the markers to be re‐introduced for analysis following mapping. In the input file, markers were ordered based on the number of missing and heterozygous lines, with markers mapping to contigs placed at the bottom of the file, so that the markers mapped to chromosomes with the largest number of informative scores would be chosen to represent the bin first. The repulsion was set to zero, so only markers scoring the same across all 120 lines or those only different due to missing data would be combined into a single bin. This resulted in 12,331 polymorphic markers being used for linkage mapping.

MapDisto2.0 (Heffelfinger et al. 2017) was used to create the linkage map. A LOD of 8 resulted in 6 Linkage Groups, with only 1 marker‐bin with 2 markers being unlinked and thus removed. Double recombinants were identified and replaced with missing data after each round of ordering/rippling.

### 2.5 QTL Analysis and Candidate Gene Identification

Quantitative Trait Loci (QTL) analysis was conducted in r/qtl (Broman et al. 2003). Composite Interval Mapping (CIM) was used with the predicted means for each environment, as well as using the combined BLUPs, representing the GEI‐independent component across all environments. Mapping parameters were set to 1 cM. One thousand permutation tests were run to determine the LOD threshold value of 4 (for a 1% significance level), which was used to declare significant QTLs.

Interactive analyses were performed over the different QTL combinations to determine additive and/or epistasis effects between significant QTLs. This was performed using the *effectplot* function of the r/qtl R package, which returned BLUP means and standard errors relative to different genotypic combinations at QTL pairs. Data were used to produce a custom plot using the ggplot2 R package (Wickham 2016).

Confidence intervals were identified using the *lodint* function of r/qtl, based on two LOD score units drop from the QTL peak. The genes between these flanked positions of the QTLs were inspected in the lentil JBrowse 2.0 (https://knowpulse.usask.ca/JBrowse2‐Lens/Lcu.2RBY).

### 2.6 *FT* Homolog Expression in a Selection of Lines Segregating for Main Flowering QTLs

Plant tissue of the parents and nine RILs segregating for the most significant DTF QTL (qDTF6.I) was harvested from block 1 at Sutherland on June 8th, 2018, ~4 weeks after sowing. Plants were sampled at the 6–8 node stage as this is close to the timepoint found to be the most useful for identifying flowering gene expression in lentils in controlled experiments (Ortega 2018). Samples were collected after sunrise, with the final plant sampled within 20 min of the first and finished before 11:00 AM. The top‐most, fully open leaf was taken from two plants and combined for each biological replication, with two biological replications harvested for each line evaluated. RNA was extracted using the Qiagen RNEasy Plant MiniKit, following the standard protocol for plant tissue. An Agilent 2100 Bioanalyzer was used to check for RNA quality and quantity.

Lines were screened for gene expression using primers for the lentil FLOWERING LOCUS *T* (*FT*) flowering gene homologs, *LcFTa1* and *LcFTb2* as well as the housekeeping gene *LcTIF* (Rajandran et al. 2022). Reverse Transcription PCR required 1 μg of RNA, 4 μL of 5× buffer, 1 μL each of dNTPs and oligo dTs, 0.5 μL Invitrogen SuperScriptTM IV Reverse Transcriptase (ThermoFischer Scientific), with dDH_2_O to make a final volume of 20 μL. The PCR protocol was a single step, with an annealing temperature of 42°C for 30 min and a cleavage step of 85°C for 5 min. The 20 μL samples of concentrated cDNA were diluted to a final volume of 100 μL.

FastSYBR Green (ThermoFischer Scientific) was used for fluorescence detection and sample setup followed the standard protocol. The qPCR program was run on a CFX 384 BioRad (ThermoFischer Scientific): 40 rounds of 95°C for 15 s, 95°C for 3 s, and 60°C for 30 s with plate fluorescence quantified every round. This procedure was followed by a melt curve analysis (as a quality check) in which the temperature was increased from 65°C to 95°C, with fluorescence quantified every 5 s as temperature increased by 0.5°C. Three technical replicates were run for each biological sample, generating six datapoints for each line. Expression of the flowering homologs (*LcFTa1, LcFTb2*) was evaluated by comparing to the expression of the housekeeping gene *LcTIF* (Actin and Translation initiation Factor, *TIF*) from the same sample using the ΔΔCt method as described by Rajandran et al. (2022).

## 3 Results

### 3.1 Phenotyping

**FIGURE 1:**
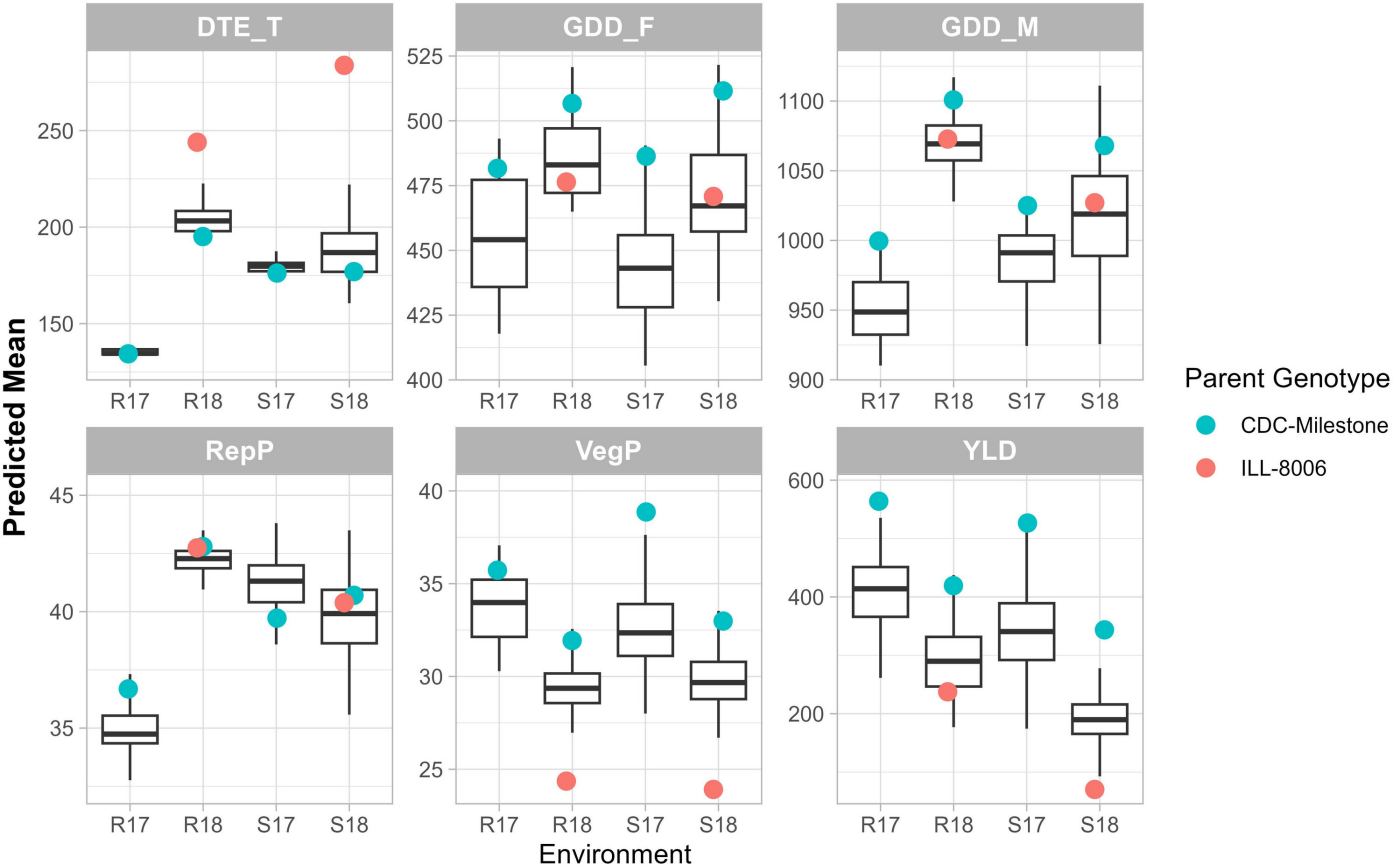
Distributions of phenotypic data for phenology‐related traits in a recombinant inbred population (LR‐11: ILL 8006 × CDC Milestone) across 4 environments; DTE_T expressed as accumulated daily temperature (°C) from sowing to emergence, growing degree days to flower (GDD_F) and maturity (GDD_M), reproductive period (RepP = DTM‐DTF), vegetative period (VegP = DTF‐DTE) and yield (YLD). Environments were Sutherland 2017 (S17), Rosthern 2017 (R17), Sutherland 2018 (S18), and Rosthern 2018 (R18).

The distributions of phenology‐related traits showed a wide variation across environments and among the RILs. The distribution across the RILs for DTE in R17 was the tightest, where the time span between the emergence of the first and last plots was only 4 days (Figure 1). Additionally, DTF, DTM, and RepP were the shortest in that late‐seeded environment. The 2017 crop season was favored by higher rainfall in addition to warmer temperatures during August in comparison with the 2018 crop season (Figure S1) which, in combination with the differences in the seeding dates, may explain the variation among environments. Notably, the 2017 crop season also produced the highest yield, with average values of 408.3 g m^−2^ and 344.6 g m^−2^ in R17 and S17, respectively. In contrast, yields in the 2018 crop season were significantly lower, with average values of 288.4 g m^−2^ in R18 and 192.2 g m^−2^ in S18 (Figure 1).

**FIGURE 2:**
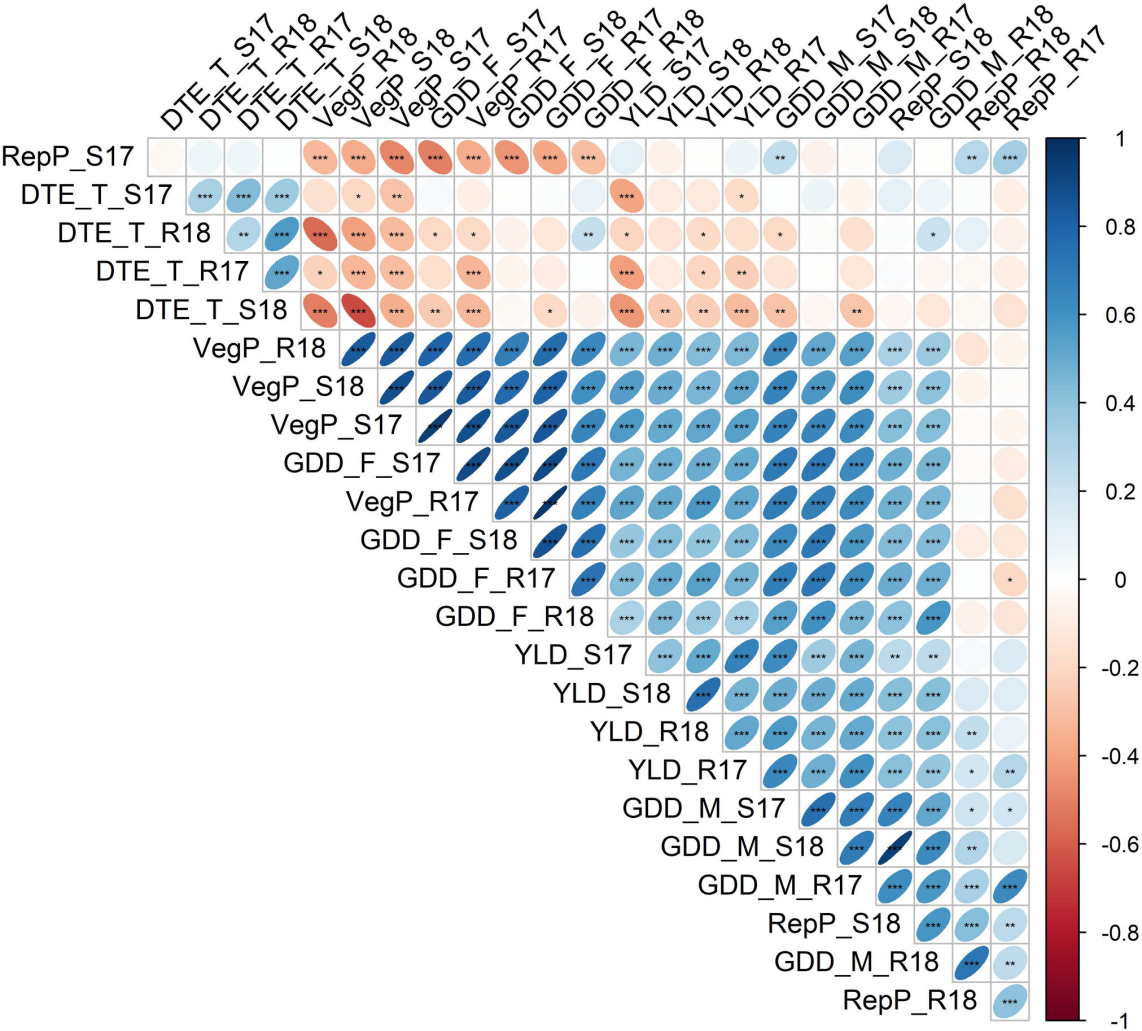
Correlation matrix for phenotypic traits measured on a recombinant inbred population (LR‐11: ILL 8006 × CDC Milestone) across four environments. Significance was determined using Pearson's correlation coefficient: Ns *p* ≥ 0.5; *p < 0.05; **p < 0.01; and ***p < 0.001.

Yield exhibited moderate to strong positive correlations with DTF, DTM, and VegP but weak correlations with RepP, except in the S18 environment (Figure 2). In addition, yield was negatively correlated with DTE in all the environments, suggesting that early emergence and an extended vegetative period positively impact yield, while prolonged reproductive periods are less beneficial (Figure 2). Interestingly, VegP demonstrated a moderate positive correlation with RepP in S18 (*r* = 0.36) but a moderate negative correlation in S17 (*r* = −0.49), further emphasizing the influence of agroclimatic variations between cropping seasons (Figure S1). Higher positive correlations were observed between VegP and DTF, even across environments (Figure 2). These traits also exhibited the highest genetic components in all environments, followed by DTM and yield (Table 1). Specifically, VegP and GDD_F showed high heritability values across environments (*H* ^2^ = 0.77 and *H* ^2^ = 0.72, respectively), making them suitable traits for breeding purposes targeting phenological adaptation in the Canadian prairies.

**TABLE 1:** Variance components of the phenology‐related traits obtained through the two‐way linear mixed model across four environments.

Parameters	DTE_T	GDD_F	GDD_M	RepP	VegP	YLD
Total variance	615.06	4.08	10.10	7.24	4.75	7244.41
Residual variance	350.39	1.02	4.28	4.26	1.14	2887.88
H^2^	0.32	0.72	0.53	0.24	0.77	0.52
$r_{i}^{2}$	0.11	0.24	0.20	0.28	0.17	0.32
$r_{\mathrm{ge}}^{2}$	0.77	0.84	0.79	0.45	0.90	0.67

*Note: H* ^2^, $r_{i}^{2}$ and $r_{\mathrm{ge}}^{2}$ represent heritability on a mean basis, the coefficient of determination of the genotype‐by‐environment effect and the genotype‐by‐environment correlation, respectively. DTE_T is expressed as accumulated average daily temperature from sowing to DTE.

Traits such as RepP and DTE_T displayed low to moderate *H* ^2^ values (0.24 and 0.32, respectively), yet a significant portion of their variation was attributed to GEI effects, as indicated by the coefficient of determination for GEI ($r_{i}^{2}$ = 0.28 and $r_{i}^{2}$ = 0.11, respectively) (Table 1). For GDD_F and GDD_M, which had *H* ^2^ values of 0.72 and 0.53, respectively, the GEI effect explained 24% and 20% of the observed variation, respectively, while the yield had the highest GEI ($r_{i}^{2}$ = 0.32).

The most stable trait across the four environments evaluated was VegP followed by GDD_F, with a genotype–environment correlation ($r_{\mathrm{ge}}^{2}$) of 0.90 and 0.84, respectively (Table 1). In contrast, the least stable trait across environments was RepP ($r_{\mathrm{ge}}^{2}$ = 0.45), followed by yield ($r_{\mathrm{ge}}^{2}$ = 0.67).

### 3.2 LR‐11 Linkage Map Construction

**FIGURE 3:**
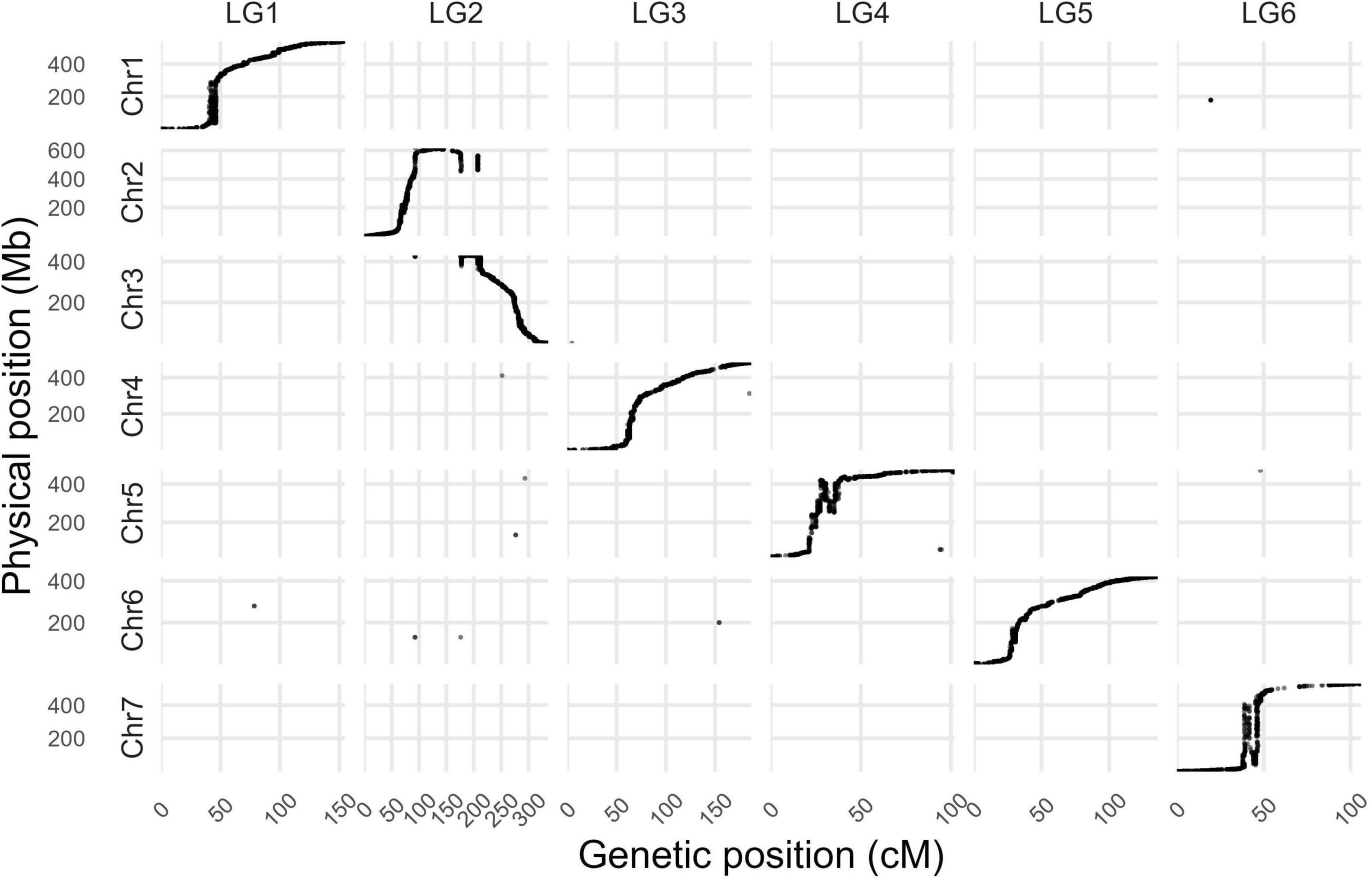
Synteny map between the physical (Mb) and the linkage (cM) map.

The LR‐11 linkage map included 12,331 single nucleotide polymorphisms (SNPs) across six linkage groups. A large group of co‐segregating markers, those associated with reference genome Lcu.2RBY chromosomes 2 (LcChr2) and 3 (LcChr 3), were pseudo‐linked so only six linkage groups (LGs) rather than the expected seven were generated (Figure 3). The clearest translocation is L2G2 × Chr3 as it does not conform to the 1:1 line in Figure 3. Other markers grouped into LGs, which match the Lcu.2RBY chromosomes, displaying collinearity as they conform to the 1:1 line in Figure 3. The complete linkage map was 1020.7 cM long with an average of 1.1 cM between uniquely mapping bins. The marker‐bin density by LG ranged from 1.33 cM per uniquely mapping bin, on LG1, to 0.82 cM per unique marker‐bin, on LG7 (Table S1). For referring to the lentil reference assembly (Lc2.RBY) Linkage Groups are presented in Table 2 as: LG1 = LcChr1, LG2 = LcChr2/3, LG3 = LcChr4, LG4 = LcChr5, LG5 = LcChr6 and LG6 = LcChr7.

**TABLE 2:** QTL summary for phenology‐related traits for the recombinant inbred population LR‐11 (ILL 8006 × CDC Milestone).

QTL block	Trait_ENV	QTL name	Linkage group	Position (cM)	LOD	Additive effect	PVE
LcChr1.I	RepP_R18	qRepP1.I	LG1	29.50	6.81	0.17	22.40
LcChr1.II	DTM_R18	qDTM1.II	LG1	46.93	4.11	0.37	15.44
	RepP_GEI	qRepP1.II	LG1	53.40	4.15	0.21	13.71
	RepP_R18	qRepP1.II	LG1	53.40	5.30	0.11	17.11
	RepP_S18	qRepP1.II	LG1	53.40	5.05	0.61	16.08
LcChr2/3.I	DTF_R18	qDTF2/3.I	LG2	11.42	4.91	−0.33	15.86
LcChr2/3.II	DTE_GEI	qDTE2/3.II	LG2	92.74	8.63	−5.05	26.12
	DTE_R17	qDTE2/3.II	LG2	92.74	7.47	−1.89	24.02
	DTE_R18	qDTE2/3.II	LG2	73.62	6.07	−3.45	22.05
	DTE_S17	qDTE2/3.II	LG2	91.79	8.56	−1.74	26.87
	DTE_S18	qDTE2/3.II	LG2	92.74	6.85	−7.62	22.59
	VegP_GEI	qVegP2/3.II	LG2	86.67	4.14	0.60	13.28
	VegP_S17	qVegP2/3.II	LG2	79.98	5.67	0.79	20.80
	YLD_GEI	qYLD2/3.II	LG2	86.67	4.69	17.10	13.75
	YLD_S17	qYLD2/3.II	LG2	87.55	9.38	19.78	29.86
LcChr2/3.III	DTE_GEI	qDTE2/3.III	LG2	176.06	8.12	−5.30	28.16
	DTE_R17	qDTE2/3.III	LG2	176.54	7.39	−1.90	24.80
	DTE_R18	qDTE2/3.III	LG2	177.41	4.59	−3.58	16.03
	DTE_S17	qDTE2/3.III	LG2	177.41	7.38	−1.62	22.02
	DTE_S18	qDTE2/3.III	LG2	180.72	7.12	−7.66	22.60
	RepP_R18	qRepP2/3.III	LG2	191.58	4.08	−0.05	14.14
	VegP_S17	qVegP2/3.III	LG2	177.88	4.59	0.86	23.19
	YLD_GEI	qYLD2/3.III	LG2	176.06	4.71	16.90	15.39
	YLD_R17	qYLD2/3.III	LG2	176.06	4.60	20.51	15.17
	YLD_S17	qYLD2/3.III	LG2	176.06	8.52	36.49	27.23
LcChr2/3.IV	DTE_GEI	qDTE2/3.IV	LG2	207.16	8.56	−5.00	26.35
	DTE_R17	qDTE2/3.IV	LG2	207.63	7.15	−1.79	21.44
	DTE_R18	qDTE2/3.IV	LG2	207.16	4.83	−3.62	18.17
	DTE_S17	qDTE2/3.IV	LG2	211.83	7.86	−1.78	25.83
	DTE_S18	qDTE2/3.IV	LG2	209.02	8.16	−7.38	27.00
	DTM_R18	qDTM2/3.IV	LG2	212.70	4.02	−0.01	8.19
	VegP_S17	qVegP2/3.IV	LG2	211.83	4.46	0.86	14.69
	YLD_GEI	qYLD2/3.IV	LG2	211.83	4.43	18.89	18.94
	YLD_R17	qYLD2/3.IV	LG2	214.51	4.10	22.30	14.56
	YLD_S17	qYLD2/3.IV	LG2	207.63	7.74	35.33	24.97
LcChr2/3.V	RepP_R18	qRepP2/3.V	LG2	282.14	4.38	0.07	15.10
LcChr4.I	RepP_R18	qRepP4.V	LG3	169.99	4.11	−0.08	16.90
LcChr5.I	YLD_S17	qYLD5.I	LG4	1.84	5.32	28.26	17.60
LcChr5.II	DTM_S17	qDTM5.II	LG4	27.91	4.35	0.54	14.88
	RepP_R17	qRepP5.II	LG4	27.00	4.03	0.20	15.71
	YLD_GEI	qYLD5.II	LG4	27.91	4.20	13.79	13.23
	YLD_S17	qYLD5.II	LG4	27.91	6.22	26.76	19.61
LcChr5.III	DTM_S17	qDTM5.III	LG4	39.46	4.34	0.62	8.84
	YLD_GEI	qYLD5.III	LG4	39.97	4.05	16.19	14.55
	YLD_S17	qYLD5.III	LG4	39.97	6.61	33.40	23.10
LcChr5.IV	DTM_S17	qDTM5.III	LG4	98.91	5.11	0.72	17.29
LcChr6.I	DTE_S18	qDTE6.I	LG5	2.34	4.51	−6.25	15.11
	DTF_GEI	qDTF6.I	LG5	7.38	17.72	0.94	46.70
	DTF_R17	qDTF6.I	LG5	7.38	22.91	1.25	55.66
	DTF_R18	qDTF6.I	LG5	7.38	7.56	0.39	21.99
	DTF_S17	qDTF6.I	LG5	7.38	16.21	1.40	44.21
	DTF_S18	qDTF6.I	LG5	8.79	10.67	0.65	31.16
	DTM_GEI	qDTM6.I	LG5	7.38	11.36	1.12	35.40
	DTM_R17	qDTM6.I	LG5	8.79	9.57	0.91	27.67
	DTM_S17	qDTM6.I	LG5	7.38	10.65	1.03	34.72
	DTM_S18	qDTM6.I	LG5	7.38	9.91	1.66	32.43
	VegP_GEI	qVegP6.I	LG5	3.25	18.57	1.18	43.54
	VegP_R17	qVegP6.I	LG5	7.38	21.81	1.28	53.52
	VegP_R18	qVegP6.I	LG5	8.79	10.62	0.73	32.36
	VegP_S17	qVegP6.I	LG5	3.25	16.58	1.53	45.60
	VegP_S18	qVegP6.I	LG5	3.25	16.10	0.98	43.16
	YLD_GEI	qYLD6.I	LG5	7.38	7.21	23.26	23.28
	YLD_R18	qYLD6.I	LG5	2.34	5.78	21.20	19.53
	YLD_S17	qYLD6.I	LG5	4.25	6.72	33.24	23.04
	YLD_S18	qYLD6.I	LG5	2.34	6.59	18.89	21.32
LcChr6.II	DTM_R18	qDTM6.II	LG5	24.30	5.14	0.98	18.57
	RepP_GEI	qRepP6.II	LG5	30.80	7.41	0.33	18.17
	RepP_R17	qRepP6.II	LG5	29.35	4.93	0.36	16.17
	RepP_R18	qRepP6.II	LG5	16.15	4.02	0.03	13.92
	RepP_S18	qRepP6.II	LG5	25.26	6.52	0.79	16.32
	YLD_R17	qYLD6.II	LG5	20.29	8.02	29.26	22.40
LcChr6.III	DTF_GEI	qDTF6.III	LG5	133.82	5.44	0.52	15.63
	DTF_R18	qDTF6.III	LG5	132.94	4.21	0.29	13.98
	DTF_S17	qDTF6.III	LG5	133.82	5.91	0.81	15.62
	DTF_S18	qDTF6.III	LG5	133.82	6.23	0.52	21.44
	VegP_GEI	qVegP6.III	LG5	133.82	4.14	0.54	10.48
	VegP_S17	qVegP6.III	LG5	132.94	4.35	0.65	17.76
LcChr7.I	YLD_S18	qYLD7.I	LG6	28.79	4.02	16.31	15.17
LcChr7.II	RepP_R18	qRepP7.II	LG6	37.92	4.51	0.07	15.92
LcChr7.III	RepP_R18	qRepP7.III	LG6	54.00	5.54	0.14	6.71

*Note:* ENV indicates in which environment the QTL reached significance. LOD is the peak logarithm of the odds score for the QTL. Percent Variation Explained (PVE) is the amount of variation explained by the locus at the peak of the QTL.

### 3.3 QTL Identification

**FIGURE 4:**
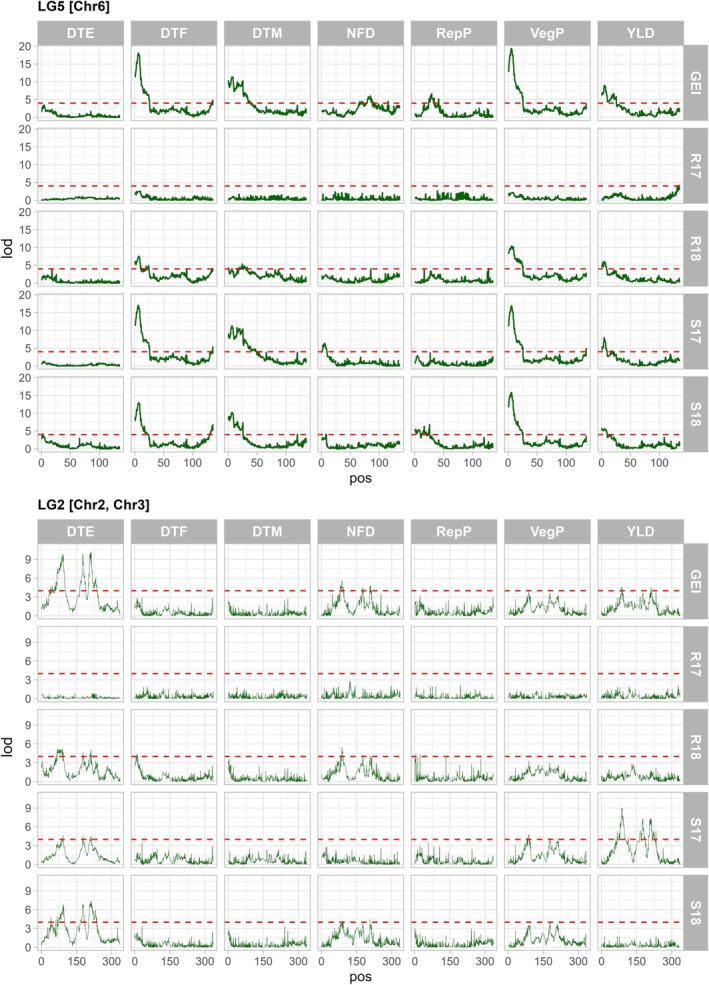
Linkage groups (LG2 top and LG5 bottom, in cM) and QTL for all the traits evaluated by each environment and across all environments (GEI). LOD score is plotted at each locus based on the BLUP of each trait. A LOD threshold of 4, shown with a dashed red line, was determined using a 1000‐permutation test (*p* < 0.01).

Phenology‐related QTLs were found on the six linkage groups (Table 2). Many of the confidence intervals for different traits overlapped when considering the CIM interval, which allowed them to be placed into QTL blocks. In total, there were 18 phenology‐related QTL groups: two on LG1, five on LG2, one on LG3, four on LG4, three on LG5 and three on LG6. The QTLs that were found in all site‐years fell into LG2 and LG5, including the QTL blocks LcChr2/3.II, LcChr2/3.III, LcChr2/3.IV and LcChr6.I. The same results were obtained for DTF and GDD_F and for DTM and GDD_M through the QTL analysis. The QTLs located in these LGs were also consistent for different traits analyzed (Figure 4). Specifically, these QTLs on LcChr6.I for VegP, DTF, and DTM explained a minimum of 32.4%, 22.0% and 17.8% of the phenotypic variation in R18. The maximum PVE for these QTLs was 53.5% (R17), 55.7% (R17) and 34.7% (S17), respectively. In addition, this QTL region explained 43.5% (VegP), 46.7% (DTF) and 35.4% (DTM) of the phenotypic variation for these same traits when mapping the GEI component generated using all four environments together, indicating this major locus has a strong effect on development of the LR‐11 RILs under Saskatchewan growing conditions.

The three peaks in the QTL blocks LcChr2/3.II, LcChr2/3.III and LcChr2/3.IV of LG2 were also consistent across environments (Table 2). QTLs for DTE, VegP, RepP, DTM and yield were found in these regions as well as LG5, although DTE was the most consistent trait across environments (Figure 4). Due to the co‐segregation of markers from LcChr2 and LcChr3, the confidence intervals for *qDTE2/3.II* are large (as much as 19 cM), even though they explain 22%–26% of the phenotypic variation in any given environment. Figure 4 highlights the relative stability of these four main loci (LcChr2/3.II, LcChr2/3.III and LcChr2/3.IV on LG2 and LcChr6.I on LG5) and plots the LOD scores using the data from each environment and across environments (GEI) for DTE, DTF, DTM, RepP, VegP, and YLD. Gross plot yield (YLD) QTLs were always located within the confidence intervals of QTLs for phenology‐related traits with major effects (Table 2).

The late emergence allele for DTE always came from the parent ILL 8006, as indicated by the negative additive genetic effect (values from −7.66 to −1.62) (Table 2). In contrast, increased VegP and DTF were derived from the adapted parent, CDC Milestone, with additive effect values from 0.50 to 1.61 and 0.46 to 1.49, respectively (Table 2).

There is an exception where the additive genetic effect is negative for DTF in the LcChr2/3.I region (qDTF2/3.I), suggesting an interaction for this trait between the locus qDTF2/3.I and the two loci on LG5 (qDTF6.I and qDTF6.II).

**FIGURE 5:**
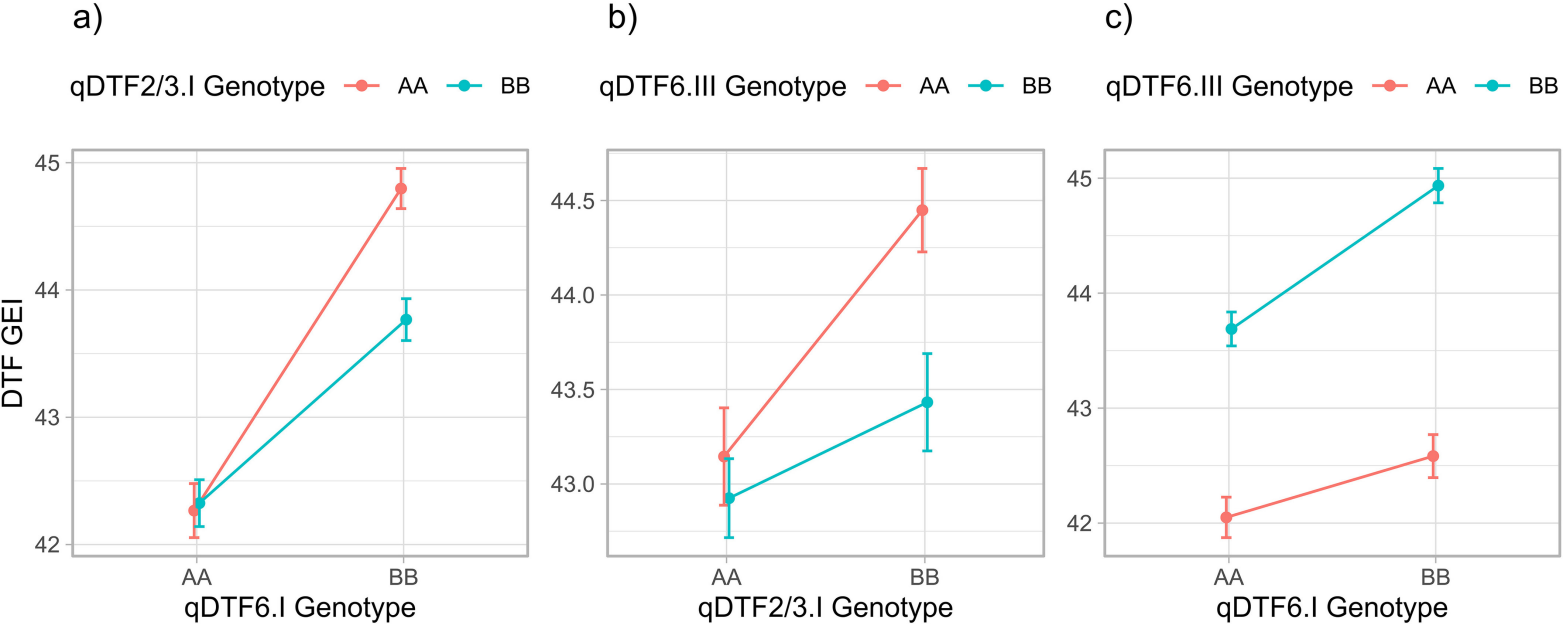
Combined effects on the phenotype of the QTL pairs *qDTF6.I*—*qDTF2/3.I* (a), *qDTF6.III*—*qDTF2/3.I* (b), and *qDTF6.III*—*qDTF6.I* (c). On the *x*‐axis is the genotype (AA or BB) at the marker locus corresponding to the first QTL peak; the color of each line/point (red or blue) indicates the genotype at the second QTL peak. The *y*‐axis shows the mean best linear unbiased predictors (BLUPs) of genotypic effects on the phenotype, with black error bars representing ± SE. Lines connect the same genotype of the second QTL to illustrate how the phenotype changes when the genotype at the first QTL varies. “AA” and “BB” denote BM4 ILL8006 and CDC Milestone alleles, respectively.

Information on the genotypic values and QTL genotypes of individual RILs was used to investigate the genetic effect of different QTL combinations in the QTL blocks LcChr2/3.I, LcChr6.II and LcChr6.I. These blocks contain QTLs for the same trait (DTF); therefore, the combinations investigated were *qDTF2/3.I*—*qDTF6.I*, *qDTF2/3.I*—*qDTF6.III* and *qDTF6.I*—*qDTF6.III* (Figure 5). In both plots (Figure 4A,B), the two genotype lines (AA vs. BB at *qDTE6.I* or *qDTF6.III*) are not parallel across the genotypes. This is evidence of an interaction effect (i.e., epistasis) rather than additivity, indicating that the effect of one QTL depends on which genotype is present at the other QTL. Conversely, the parallel lines in Figure 5c suggest an additive effect because each locus contributes the same shift in DTF, regardless of the allele at the other locus.

### 3.4 Candidate Gene Identification

To identify candidate genes underlying the QTLs associated with DTE and DTF in lentil, we examined the genomic regions within major QTL blocks on linkage groups LG2 and LG5, which correspond to chromosomes Chr2/Chr3 and Chr6, respectively.

The QTL block LcChr2/3.I on LG2 harbored a QTL for DTF (*qDTF2/3.I*), unique as the only DTF QTL outside LG5 in this study. Its peak marker resides within the gene *Lcu.2RBY.2 g001730*, encoding a ribosome biogenesis regulatory protein (Table S2). The confidence interval was quite small for this locus, and this was the only gene in the interval.

Three additional QTL blocks on LG2 (LcChr2/3.II, LcChr2/3.III, and LcChr2/3.IV) consistently contained QTLs for DTE across all site‐years. The peak markers for these 12 DTE QTLs mapped to Chr2, spanning a physical distance of approximately 84 Mb (398,818,389 to 482,878,377 bp). This large interval, possibly reflecting low recombination or mapping complexity due to LG2 being the result of pseudolinkage of markers from both Chr2 and Chr3, encompasses six curated genes in the CDC Redberry v2.0 genome linked to plant growth and development: Two Squamosa Promoter Binding‐Like (*SPL*) genes (*LcuSPL6c* and *LcuSPL1c*); an ARIA‐Interacting Double AP2 Domain Protein (*ADAP*) gene (*LcADAP2*); an *E1*‐like gene (*LcE1*); an Argonaute‐like (*AGO*) gene (*LcAGO1/10*) and an Altered Phloem Development (*APL*) gene (*LcFE*) (Table S2). These genes, particularly *SPLs* and *E1*‐like, are plausible candidates for regulation of DTE due to their established roles in developmental timing in other species.

The QTL block LcChr6.I (LG5), located at the top of Chr6, contained major QTLs for all phenology‐related traits except RepP, spanning a genetic distance of 2.34 to 8.79 cM (approximately 4.5 Mb; 61,101 to 4,590,974 bp). A key candidate within this block is the *FTb1/FTb2* gene, a homolog of FLOWERING LOCUS T (*FT*), strongly implicated in the promotion of flowering under long‐day conditions in legumes, including lentils (Hecht et al. 2011; Haile et al. 2021; Neupane et al. 2022). *In silico* analysis further identified additional flowering‐related genes (Table S2), including: *Lcu.2RBY.6 g000570*, involved in trehalose metabolism, which supports flowering induction, and *Lcu.2RBY.6 g000590*, associated with flowering delay via proteostasis regulation (Ionescu et al. 2016; Blanco‐Touriñán et al. 2021).

A second QTL block on LG5, LcChr6.III, spans a narrow genetic distance of 0.88 cM (from 132.94 to 133.82 cM) and includes a DTF QTL detected in three of four environments (not R17). This block encompasses markers from two distinct physical regions on Chr6: a proximal segment (196,688 to 298,283 bp; 101.6 Kb) and a distal segment (419,817,975 to 420,429,777 bp; 611.8 Kb). These two distant regions in the physical map may exhibit elevated recombination rates and/or linkage disequilibrium, explaining why they cluster together in the linkage group 5. The proximal region harbors five genes, while the distal region contains 42, including the curated genes: *LcPRR59c* and *LcLHY*, circadian clock components that regulate flowering time; as well as *LcDCL1*, involved in microRNA biogenesis, influencing flowering via gene regulation (Rajandran et al. 2022; Ridge et al. 2017). These genes, particularly those in the distal region, are likely candidates for DTF variation due to their roles in photoperiod and developmental pathways.

### 3.5 Expression of Candidate Genes for DTF in LR‐11

**FIGURE 6:**
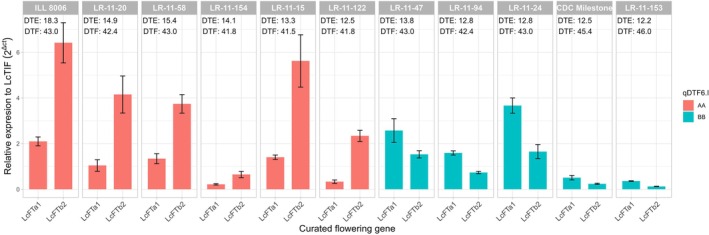
Relative RNA expression to *LcTIF* of *LcFTa1* and *LcFTb2* genes from leaf tissue in a subset of LR‐11 lines grouped by allele state at qDTF6.I. From right to left, lines are sorted by ascending VegP (DTF‐DTE) values: LR‐11‐153, the highest and ILL 8006, the lowest. AA (red) is homozygous for the ILL 8006 allele; BB (blue) is homozygous for the CDC Milestone allele.

To explore the nature and role of qDTF6.1, its effect on gene expression was examined in two groups of RILs with contrasting qDTF6.1 genotype. Relative expression levels of the main positional candidate *LcFTb2* were determined in leaf tissue taken from four‐week‐old plants, normalized to *LcTIF* gene expression. Figure 6 shows that lines carrying the early (i.e., ILL 8006) allele of qDTF6.1 express *LcFTb2* at a significantly (*p* < 0.001) higher level than those carrying the late allele. This result is consistent with a scenario in which the effect of qDTF6.1 on flowering time reflects a genetic difference at or near the *FTb2* gene that influences its expression.

We also examined expression of a second *FT* gene, *LcFTa1*, in this material. Although *FTa1* is located in the central region of Chr6, where no QTL was detected in this study, it has been reported as another key regulator of flowering time in legumes (Laurie et al. 2011; Hecht et al. 2011; Ortega et al. 2019), including lentil (Rajandran et al. 2022). However, there was no clear effect of qDTF6.1 on *FTa1* expression.

## 4 Discussion

### 4.1 Phenology‐Related Trait Variation in LR‐11 Population

We investigated phenology‐related traits expressed by RILs from a cross between a South Asian line, ILL 8006, and CDC Milestone, an adapted western Canadian cultivar. South Asian germplasm typically flowers incredibly early in western Canada due to the long days and mild temperatures experienced when planted there in the spring (Wright et al. 2020). In addition to the expected variability in DTF, we also noted variability in DTE with alleles from the South Asian parent contributing to later emergence. The contrasting developmental reactions of the parents to the western Canadian environment make the LR‐11 RIL population an effective method of studying the genetic and environmental requirements that influence vegetative period and flowering time in the region.

Heritability (*H* ^2^) estimates based on variance components (Table 1) indicate moderate to high heritability for most of the phenology‐related traits in this study, with the exception of RepP, consistent with other long‐day field studies in lentils (Haile et al. 2021; Tullu et al. 2008). This is indicative of a large genetic component to phenotypic variation, which justifies the use of these traits for QTL mapping. The consistency of trait relationships across environments gives a strong basis for investigating the genomic regions underlying these traits. Vegetative phase (VegP) and reproductive phase (RepP) were also calculated and used as phenological traits. They have conventionally been applied in lentil research to differentiate plant growth traits, especially those related to environmental effects on flowering and maturity (Wright et al. 2020; Roberts et al. 1988). Gross plot seed yield (YLD) was highly correlated with all phenology‐related traits in most environments, indicating the importance of flowering time as an effective selection criterion for yield improvement as has previously been reported in the area (Fedoruk et al. 2013; Tullu et al. 2010). Notably, longer VegP and later DTF were most highly correlated with greater YLD. Early emergence (i.e., low DTE) also always correlated with greater yields in most site‐years, emphasizing the need for an extended period of vegetative growth to support higher seed yields.

Conversely, RepP had a low heritability on a mean basis (across environments) in this study and is hence not a suitable candidate for QTL identification nor selection in a breeding program. This was also found by Wright et al. (2020), who observed lower variation in RepP among accessions and environments of a lentil diversity panel grown in comparison to VegP across a wide range of conditions, suggesting that it is the VegP and not the RepP driving adaptation in lentil. Therefore, developmental stages before flowering appear more important for suitable agronomic performance.

### 4.2 VegP Is Under Control of Two Different Growing Phases: Emergence and Flowering

Statistically significant genetic and genetic‐by‐environmental terms were detected in the linear mixed model for DTE. These results support treating DTE as an independent trait for analysis, adding a complementary dimension to understanding the DTF differences between the LR‐11 parents and among the RILs across different environments. The early‐flowering parent, ILL 8006, emerged ~7 days later than CDC Milestone at both Sutherland and Rosthern in 2018 but compensated with a markedly reduced VegP (~11 days shorter at both sites), resulting in earlier flowering. When considering DTE and VegP together (DTF), no transgressive segregation was observed among the RILs: CDC Milestone exhibited the earliest emergence and longest VegP, while ILL 8006 showed the latest emergence and shortest VegP (Figure 1). Within‐environment analyses revealed a negative correlation between DTE and VegP across the RILs (Figure 2), mirroring the parental trend where later‐emerging lines tended to have shorter VegP. In Saskatchewan, growth habits like CDC Milestone (early emergence, late flowering) are preferred to maximize the vegetative period and support higher yields. However, RILs combining early emergence (CDC Milestone‐like) with hastened flowering alleles (ILL 8006‐like) could be valuable for other lentil‐growing regions such as South Asia, resulting in lines with maximum VegP under short daylength growing seasons. In such cases, reliable markers for both DTE and DTF would be beneficial. Although there are no previous reports on ILL 8006 earliness in western Canada locations, multi‐environmental trials on the parent ILL 5888 showed that it has abnormally elevated temperature sensitivity and photoperiod insensitivity (Wright et al. 2020). This could explain the flowering earliness of ILL 8006 during summer season in 2018 when it was grown in Southerland and Rosthern (Figure 1). DTE variability was much smaller in the late‐seeded environment (R17), suggesting it could be related to soil temperature. Cool soil temperature is not common in South Asian growing conditions since they seed in the autumn when the soils are still warm, eliminating differences in emergence due to soil temperature. The lack of correlation between DTE and DTF, contrasted with the consistent negative correlation between DTE and VegP, alongside the lower DTE heritability (0.32) compared with the higher heritability of VegP (0.77) and DTF (0.72), may reflect environmental variables. There is only one environment, S17, where qVegP is collocated with qDTE: in the LcChr2/3.II, LcChr2/3.III and LcChr2/3.IV blocks. In addition, there was only one environment, S18, where qDTE co‐located with qDTF in the LcChr6.I block. In contrast, all the QTLs detected for DTF and VegP always co‐located. This suggests that genes controlling flowering time have a stronger influence on VegP and that the DTE genetic component is independent of other traits. DTE may modulate DTF (and consequently, VegP); however, under certain environmental conditions, as in S17 and S18.

The large confidence intervals for qDTE on LG2, compounded by what is likely a translocation in ILL 8006 relative to both CDC Milestone and the CDC Redberry reference line, complicate candidate gene searches, with significant markers spanning wide regions of LcChr2 and LcChr3. Translocations lead to reduced recombination and increased linkage drag in chromosomes that are involved and hinder precise selection.

### 4.3 Linkage and QTL Mapping of Phenological Traits

The linkage map constructed for the LR‐11 population, based on 12,331 uniquely mapping exome‐capture‐derived SNP markers, represents one of the denser bi‐parental lentil linkage maps to date. Its total map length is comparable to other SNP‐based maps derived from cultivated parents (Ates et al. 2016, 2018; Haile et al. 2021). Despite the high marker density, the conservation of markers beyond mapping populations remains a recognized challenge, limiting their broader applicability (Kumar et al. 2021; Tullu et al. 2010). To address this, marker utility across diverse lentil germplasm has been ensured since only markers that were also present in the AGILE‐LDP genotyping results (Haile et al. 2020) were retained, facilitating their integration into the ongoing lentil breeding programs.

Different settings of marker clean‐up and linkage map construction parameters (not shown) consistently yielded 6 linkage groups, as opposed to the 7 expected based on lentil having 7 chromosome pairs (Figure 3). These results suggest that a translocation involving the exchange of segments between two non‐homologous chromosomes, LcChr2 and LcChr3, exists in ILL 8006 relative to CDC Milestone. Similar discrepancies have been reported in other lentil linkage maps due to a translocation in one parent relative to the other (Ramsay et al. 2021; Yuan et al. 2021; Cao et al. 2024). Such issues are common in populations involving crosses between cultivated and wild *Lens* spp., as found by Cao et al. (2024) in three interspecific lentil RILs (LR‐68, LR‐70, and LR‐86). The finding of a similar pattern of pseudolinkage points to a translocation within the cultivated species. The markers used in this map are associated with genes in the Lcu.2RBY genome and, as such, it is possible to refine candidate gene identification based on that genome.

Despite the translocation, the peak markers for DTE QTLs in LcChr2/3.II, LcChr2/3.III, and LcChr2/3.IV consistently map to LcChr2. This suggests that the genetic factors influencing DTE are located on LcChr2, and the translocation does not prevent their identification in the linkage map. This finding is particularly interesting given the complexity introduced by the translocation, which typically increases linkage drag and broadens QTL confidence intervals (Farré et al. 2012). Therefore, the fact that no peak markers map to LcChr3 suggests that LcChr3 segments in LG2 do not harbor significant genetic variation for DTE in the LR‐11 population, or that the markers on LcChr3 are less informative for these QTLs. Rajandran et al. (2022) also identified two QTLs for DTE in lentil, supporting the idea that the period between sowing and emergence is controlled by genomic regions distinct from those regulating the time from emergence to flowering (VegP). However, their QTLs, located on linkage groups 5 and 7, appear to differ from those identified here. This discrepancy might be explained by the use of different genetic backgrounds, as the population analyzed by Rajandran et al. (2022) did not include South Asian accessions.

Within the 84 Mb region on LcChr2, six curated genes with a possible connection to seed germination and seedling emergence were identified. *LcSPL6c* and *LcSPL1b* belong to the well‐known family of SPL transcription factors that are regulated by miR156 and play key roles in developmental transitions, including flowering time and phase changes (Xu et al. 2016). Recent studies have also linked *SPL* genes to seed development and germination. For instance, in *Oryza sativa* , *SPL12* influences seed size and seed dormancy by delaying germination (Qin et al. 2020), and overexpression of different SPL genes has shown stable delayed flowering time in alfalfa ( *M. sativa* ) and soybean ( *G. max* ) (Cao et al. 2015; Ma et al. 2022).

*LcAGO1/10* is a member of the Argonaute‐like gene family, which encodes components of the RNA‐induced silencing complex and influences gene expression via small RNAs too. *AGO1* is essential for miRNA‐mediated regulation during seedling development in *Arabidopsis thaliana* (Tognacca and Botto 2021). *AGO*‐like genes are also involved in seed dormancy. For instance, an *AGO4*‐like gene in barley ( *Hordeum vulgare* ) is a negative translation regulator that affects the expression of dormancy genes (Singh and Singh 2012). In addition, recent studies have found that members of the *AGO* family interact with *SPL* genes to control developmental timing (Hoyer et al. 2019; Roussin‐Léveillée et al. 2020).

The ARIA‐Interacting Double AP2 Domain Protein (*LcADAP2*) encodes a transcription factor involved in developmental signaling. *ADAP*‐like genes are expressed in roots, emerging young leaves, and flowers. A study from Lee et al. (2009) found that *ADAP* knockout mutant lines germinate more efficiently than wild‐type plants in *A. thaliana* . In addition, mutant seedlings grow faster, suggesting that *ADAP* participates in the regulation of germination and seedling growth (Lee et al. 2009).

The *LcE1* gene is homologous to the soybean ( *G. max* ) *E1* gene, a major regulator of flowering time under long‐day conditions (Xia et al. 2012). Although primarily associated with flowering, some flowering time genes exhibit pleiotropic effects on other developmental stages (Cheng et al. 2020; Takeshima et al. 2019). For instance, *FT5a* in soybean has a dual function in the regulation of post‐flowering stem growth and flowering time (Takeshima et al. 2019). Candidate genes for DTE in legumes, such as those responsive to growth regulators and temperature changes, likely influence development beyond emergence (Dias et al. 2010; Weller and Ortega 2015). Nonetheless, the roles of *E1* in other developmental processes, such as seed germination or dormancy, are unknown, warranting further investigation in this direction.

The last curated candidate gene for DTE corresponds to the *LcFE* gene, which encodes an Altered Phloem Development (*APL*) gene. This is a *MYB* transcription factor essential for phloem differentiation in *A. thaliana* (Bonke et al. 2003). An *APL1*‐like gene was found to be expressed at elevated levels during flowering in olive trees ( *Olea europaea* ) and was also expressed during the cold incubation activating embryo germination, suggesting a probable role in embryonic development (Jiménez‐Ruiz et al. 2018). Therefore, while not directly involved in seed germination, the role of APL in vascular development makes it a candidate for influencing DTE through post‐germination growth dynamics.

The most significant QTL for days to flowering (DTF), designated qDTF6.I, was identified on LG5. This QTL co‐localizes with other phenology‐related traits and features a peak dominated by markers within a 0.6–4.6 Mbp region on LcChr6. In this interval, there are two remarkably similar lentil homologues of *FTb*, *LcFTb1* and *LcFTb2*, that participate in photoperiod regulation in pea plants under long‐day conditions (Hecht et al. 2011). Under Saskatchewan field conditions, where lentils receive long days (> 12 h) after emergence, loss of photoperiod flowering control is most likely to speed up flowering. This same region was important for controlling DTF in another lentil population with a different un‐adapted parent (ILL 1704) (Haile et al. 2021) as well as a diversity panel (Neupane et al. 2022) grown in Saskatchewan.

Expression analysis of *LcFTb2* in a subset of LR‐11 RILs revealed higher transcript levels in lines carrying the ILL 8006 (early) allele compared with those with the CDC Milestone (late) allele (Figure 6). Similarly, early flowering mutants in pea exhibit elevated *FTb2* expression under long‐day requirements (Ridge et al. 2016; Hecht et al. 2011). There are other candidate genes within this region; however, that deserve further investigation (Table S2). For instance, *Lcu.2RBY.6g000570* encodes a “sweetie” protein involved in sugar metabolism in *M. truncatula* and *A. thaliana* (Ionescu et al. 2016; Cheng et al. 2020). In *A. thaliana* , the *sweetie* mutant accumulates up to four times higher trehalose levels than the wild type (Veyres et al. 2008). Trehalose is known to induce *FT* expression, linking photoperiod and carbohydrate signaling to flowering control (Wahl et al. 2013). Another putative candidate gene within this QTL region, *Lcu.2RBY.6g000590*, encodes a T‐complex protein, which, alongside prefoldins (PFDs), contributes to proteostasis and flowering time regulation. Blanco‐Touriñán et al. (2021) showed that PFDs postponed flowering by downregulating the expression of integrator genes, a process supported by the observed delayed flowering among genotypes carrying the CDC Milestone allele in qDTF6.I, while the ILL 8006 allele induces earliness.

The qDTF6.III QTL mapped to two intervals on the Lcu.2RBY assembly (0.19–0.29 Mbp and 419.81–420.43 Mbp), suggesting that a large structural rearrangement, for example, a translocation or inversion, may exist in one of the LR‐11 mapping population parents relative to the reference assembly. This may place two distant regions of chromosome 6 into close genetic linkage, resulting in a single QTL. The “distal” qDTF6.III region (at ≈420 Mb) coincides with a QTL for DTF previously identified in a controlled‐environment study under short photoperiods by Rajandran et al. (2022), where the early allele also derived from a South Asian landrace (ILL2601). Examination of candidates in this region identified a potential frameshift mutation in the LcPRR59c gene in ILL2601 (Rajandran et al. 2022), but no further functional examination of this polymorphism has been reported.

In this work, we have dissected the phenology of lentils of a bi‐parental RIL population into distinct phases—emergence, flowering, and maturity—to be able to calculate vegetative and reproductive periods. This addresses one of the largest challenges in lentil breeding in temperate regions with summer growing seasons: maladaptation of varieties to long photoperiods will cause flowering to start early and cut short the vegetative period, resulting in reduced yield. While previous research had already identified lentil flowering time genes, this work is the first to examine flowering and vegetative phase under the influence of variability in days to emergence. We mapped QTLs for DTE and DTF and other derived traits in this population, enabling us to propose candidate genes controlling DTE in lentil for the first time. Our findings indicate that selection for early emerging lentil varieties can promote longer vegetative phases, thereby raising yield potential. We provide insights into the genetic regulation of emergence and its consequences on flowering. Although further analysis is required for gene validation, this research offers practical implications for breeding programs to optimize lentil adaptation and productivity under long‐day conditions.

## Author Contributions

S.O.‐C.: data analyses; writing second draft. T.H.: Investigation; data collection and analyses; writing first draft. J.L.W.: Conceptualization; Methodology; Supervision; Writing – review and editing. K.E.B.: Conceptualization; Funding acquisition; Investigation; Methodology; Project administration; Resources; Supervision; Writing – review and editing.

## Funding

This work was supported by the “Application of Genomics to Innovation in the Lentil Economy (AGILE),” project funded by Genome Canada and managed by Genome Prairie. We are grateful for the matching financial support from Western Grains Research Foundation, Saskatchewan Pulse Growers, the Government of Saskatchewan, and the University of Saskatchewan.

## Supporting information


**Data S1:** Supporting Information.

## Data Availability

All data generated and/or used in this study can be found at https://knowpulse.usask.ca/research‐study/Lens‐flowering‐LR11‐LDP.
