# Supplementary material for: Dissecting Vegetative Period Into Its Phenotypic and Genotypic Components Allows Environment‐Specific Breeding in Lentil ( Lens culinaris Medik)
Source: Physiol Plant. 2025 Dec 29;178(1):e70729. doi: 10.1111/ppl.70729 (PMC12746221; doi:10.1111/ppl.70729)
Supplement: Supplementary file 1 — Data S1: Supporting Information. [file PPL-178-e70729-s001.pdf]

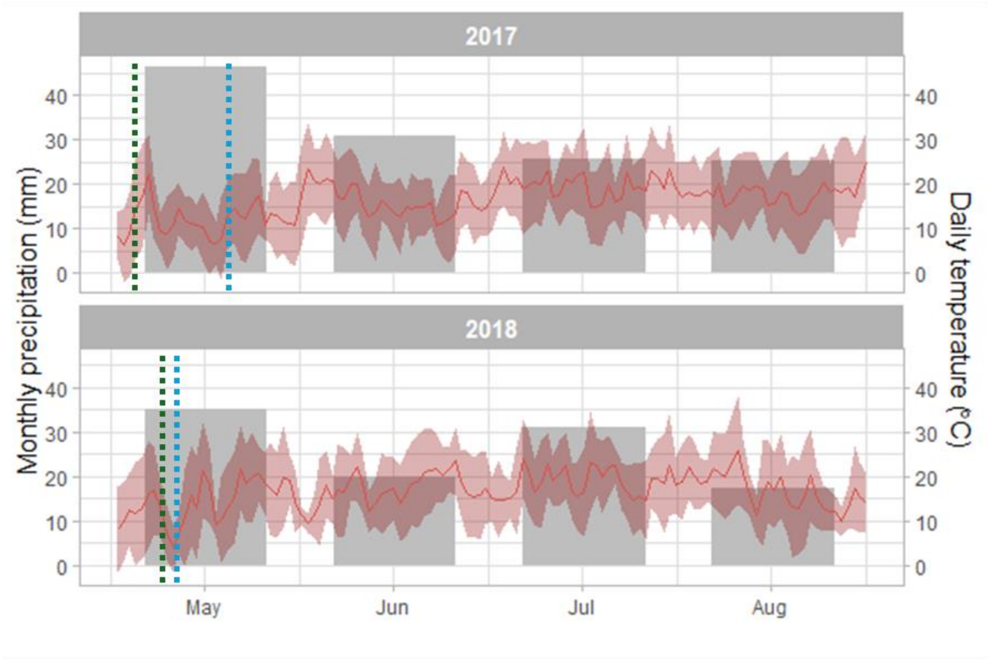

Supplementary Figure 1. Climatic variables in 2017 and 2018 crop seasons. Daily mean temperature (red line) from May to August. The shaded red area represents the min and max daily temperature while the grey bars depict the monthly accumulated precipitation. Blue and green dashed lines represent the seeding date of Southerland and Rosthern, respectively.

Supplementary Table 1. Linkage map summary for the recombinant inbred population LR-11 (ILL 8006 x CDC Milestone). Data is presented both for the entire map (LR-11) but is also presented based on each linkage group (LG). number of markers (N markers), number of unique marker-bins (N unique), linkage group size in centimorgans (cM), maximum distance between two linked markers in cM (Max cM), median distance between two linked markers in cM (Med cm), maximum number of markers mapping to a single bin (Max Dup), and median number of markers mapping to a single bin (Med Dup) are presented.

Linkage Group	N markers	N unique	cM	Max cM	Med cM	Max Dup	Med dup
**LG1**	1863	230	154.58	25.99	0.52	228	3
**LG2**	4366	490	335.46	31.08	0.50	156	2
**LG3**	2039	267	186.78	14.49	0.50	82	3
**LG4**	1237	160	102.02	17.32	0.51	173	2
**LG5**	1645	238	135.82	40.69	0.49	87	3
**LG6**	1181	147	106.01	5.34	0.49	389	3
LR-11	12331	1532	1020.7	40.69	0.50	389	3

Supplementary Table 2. *QTL plus candidate putative genes for the phenology-related traits in the LR-11 population.*

n	QTL_block	Linkage _Group	Trait	QTL name	Peak_Marker	Position _cM	Left_Marker	Right_Marker	LOD	Additive_ Effect	PVE	Candidate Putative Gene in the QTL block interval
1	LcChr1.I	LG1	RepP_R18	*qRepP1.I*	SNP_008572265	29.50	SNP_008557228	SNP_009320643	6.81	0.17	22.40	*Lcu.2RBY.1g003150*
2	LcChr1.II	LG1	DTM_R18	*qDTM1.II*	SNP_299288091	46.93	SNP_299288082	SNP_294094059	4.11	0.37	15.44	
3		LG1	RepP_R18	*qRepP1.II*	SNP_340536928	53.40	SNP_344038290	SNP_340885296	5.30	0.11	17.11	*Lcu.2RBY.1g042520*
4		LG1	RepP_GEI	*qRepP1.II*	SNP_340536928	53.40	SNP_344038290	SNP_340885296	4.15	0.21	13.71	
5		LG1	RepP_S18	*qRepP1.II*	SNP_340536928	53.40	SNP_344038290	SNP_340885296	5.05	0.61	16.08	
6	LcChr2/3.I	LG2	DTF_R18	*qDTF2/3.I*	SNP_002843767	11.42	SNP_002825316	SNP_003023060	4.91	-0.33	15.86	*Lcu.2RBY.2g001730*
9	LcChr2/3.II	LG2	DTE_R18	*qDTE2/3.II*	SNP_189091037	73.62	SNP_188567823	SNP_187937598	6.07	-3.45	22.05	*Lcu.2RBY.2g063470*
10		LG2	DTE_GEI	*qDTE2/3.II*	SNP_425141363	92.74	SNP_424815371	SNP_425141399	8.63	-5.05	26.12	*Lcu.2RBY.3g073900*
11		LG2	DTE_R17	*qDTE2/3.II*	SNP_469655104	92.74	SNP_462870458	SNP_469823003	7.47	-1.89	24.02	*Lcu.2RBY.2g063820*
12		LG2	DTE_S17	*qDTE2/3.II*	SNP_447184061	91.79	SNP_446238784	SNP_449099292	8.56	-1.74	26.87	*Lcu.2RBY.2g063930*
13		LG2	DTE_S18	*qDTE2/3.II*	SNP_453428850	92.74	SNP_452665286	SNP_454147545	6.85	-7.62	22.59	*Lcu.2RBY.2g065430*
14		LG2	VegP_GEI	*qVegP2/3.II*	SNP_396678480	86.67	SNP_404720357	SNP_396979905	4.14	0.60	13.28	*Lcu.2RBY.2g034940*
15		LG2	VegP_S17	*qVegP2/3.II*	SNP_329688588	79.98	SNP_324609920	SNP_330132877	5.67	0.79	20.80	
16		LG2	YLD_GEI	*qYLD2/3.II*	SNP_396678480	86.67	SNP_404720357	SNP_396979905	4.69	17.10	13.75	
17		LG2	YLD_S17	*qYLD2/3.II*	SNP_406898931	87.55	SNP_408223748	SNP_408418084	9.38	19.78	29.86	
18	LcChr2/3.III	LG2	DTE_GEI	*qDTE2/3.III*	SNP_467145886	176.06	SNP_453077332	SNP_511441606	8.12	-5.30	28.16	*Lcu.2RBY.2g065070*
19		LG2	DTE_R17	*qDTE2/3.III*	SNP_457036313	176.54	SNP_457036289	SNP_464748487	7.39	-1.90	24.80	*Lcu.2RBY.2g072360*
20		LG2	DTE_R18	*qDTE2/3.III*	SNP_482878377	177.41	SNP_464748487	SNP_485925272	4.59	-3.58	16.03	*Lcu.2RBY.2g071030*
21		LG2	DTE_S17	*qDTE2/3.III*	SNP_400210990	177.41	SNP_399181898	SNP_400375533	7.38	-1.62	22.02	*Lcu.2RBY.2g063200*
22		LG2	DTE_S18	*qDTE2/3.III*	SNP_426148001	180.72	SNP_426147222	SNP_426148179	7.12	-7.66	22.60	
23		LG2	RepP_R18	*qRepP2/3.III*	SNP_429102752	191.58	SNP_428519710	SNP_429213696	4.08	-0.05	14.14	
24		LG2	VegP_S17	*qVegP2/3.III*	SNP_425857180	177.88	SNP_425838690	SNP_425922141	4.59	0.86	23.19	
25		LG2	YLD_GEI	*qYLD2/3.III*	SNP_130074067	176.06	SNP_130020663	SNP_130151711	4.71	16.90	15.39	
26		LG2	YLD_R17	*qYLD2/3.III*	SNP_130074067	176.06	SNP_130020663	SNP_130151711	4.60	20.51	15.17	
27		LG2	YLD_S17	*qYLD2/3.III*	SNP_130074067	176.06	SNP_130020663	SNP_130151711	8.52	36.49	27.23	
28	LcChr2/3.IV	LG2	DTE_GEI	*qDTE2/3.IV*	SNP_463948190	207.16	SNP_463704117	SNP_463955496	8.56	-5.00	26.35	*Lcu.2RBY.2g066880*
29		LG2	DTE_R17	*qDTE2/3.IV*	SNP_463959829	207.63	SNP_463947476	SNP_470213904	7.15	-1.79	21.44	*Lcu.2RBY.2g070750*

30		LG2	DTE_R18	*qDTE2/3.IV*	SNP_463948190	207.16	SNP_463704117	SNP_463955496	4.83	-3.62	18.17	*Lcu.2RBY.2g064640*
31		LG2	DTE_S17	*qDTE2/3.IV*	SNP_398818389	211.83	SNP_398343059	SNP_398818631	7.86	-1.78	25.83	
32		LG2	DTE_S18	*qDTE2/3.IV*	SNP_417991758	209.02	SNP_416302642	SNP_420690382	8.16	-7.38	27.00	
34		LG2	DTM_R18	*qDTM2/3.IV*	SNP_359764185	212.70	SNP_358774673	SNP_360058587	4.02	-0.01	8.19	
35		LG2	VegP_S17	*qVegP2/3.IV*	SNP_362806396	211.83	SNP_362805140	SNP_362980891	4.46	0.86	14.69	
36		LG2	YLD_GEI	*qYLD2/3.IV*	SNP_405227802	211.83	SNP_398818631	SNP_412411033	4.43	18.89	18.94	
37		LG2	YLD_R17	*qYLD2/3.IV*	SNP_349651515	214.51	SNP_349160537	SNP_354810150	4.10	22.30	14.56	
38		LG2	YLD_S17	*qYLD2/3.IV*	SNP_463947476	207.63	SNP_416316987	SNP_463959829	7.74	35.33	24.97	
39	LcChr2/3.V	LG2	RepP_R18	*qRepP2/3.V*	SNP_100134466	282.14	SNP_100134412	SNP_10016449	4.38	0.07	15.10	
40	LcChr4.I	LG3	RepP_R18	*qRepP4.V*	SNP_471187393	169.99	SNP_471060457	SNP_471223950	4.11	-0.08	16.90	
41	LcChr5.I	LG4	YLD_S17	*qYLD5.I*	SNP_021904583	1.84	SNP_021131011	SNP_022323027	5.32	28.26	17.60	
42	LcChr5.II	LG4	RepP_R17	*qDTM5.II*	SNP_312178027	27.00	SNP_308764179	SNP_315750087	4.03	0.20	15.71	
43		LG4	DTM_S17	*qRepP5.II*	SNP_419101609	27.91	SNP_418999318	SNP_419181155	4.35	0.54	14.88	
44		LG4	YLD_GEI	*qYLD5.II*	SNP_419101609	27.91	SNP_418999318	SNP_419181155	4.20	13.79	13.23	
45		LG4	YLD_S17	*qYLD5.II*	SNP_419101609	27.91	SNP_418999318	SNP_419181155	6.22	26.76	19.61	
46	LcChr5.III	LG4	DTM_S17	*qDTM5.III*	SNP_429220827	39.46	SNP_428315051	SNP_432233386	4.34	0.62	8.84	*Lcu.2RBY.5g059880*
47		LG4	YLD_GEI	*qYLD5.III*	SNP_431258438	39.97	SNP_429220827	SNP_432233386	4.05	16.19	14.55	*Lcu.2RBY.5g059930*
48		LG4	YLD_S17	*qYLD5.III*	SNP_431258438	39.97	SNP_429220827	SNP_432233386	6.61	33.40	23.10	
49	LcChr5.IV	LG4	DTM_S17	*qDTM5.III*	SNP_473181008	98.91	SNP_473091703	SNP_473187971	5.11	0.72	17.29	
50	LcChr6.I	LG5	DTE_S18	*qDTE6.I*	SNP_004590974	2.34	SNP_004590635	SNP_004590997	4.51	-6.25	15.11	*Lcu.2RBY.6g000730*
51		LG5	DTF_GEI	*qDTF6.I*	SNP_001630734	7.38	SNP_001042170	SNP_001633487	17.72	0.94	46.70	*Lcu.2RBY.6g000760*
52		LG5	DTF_R17	*qDTF6.I*	SNP_001630734	7.38	SNP_001042170	SNP_001633487	22.91	1.25	55.66	
53		LG5	DTF_R18	*qDTF6.I*	SNP_001630734	7.38	SNP_001042170	SNP_001633487	7.56	0.39	21.99	
54		LG5	DTF_S17	*qDTF6.I*	SNP_001630734	7.38	SNP_001042170	SNP_001633487	16.21	1.40	44.21	
55		LG5	DTF_S18	*qDTF6.I*	SNP_001042170	8.79	SNP_00965514	SNP_001633487	10.67	0.65	31.16	
56		LG5	DTM_GEI	*qDTM6.I*	SNP_001630734	7.38	SNP_001042170	SNP_001633487	11.36	1.12	35.40	
58		LG5	DTM_R17	*qDTM6.I*	SNP_001042170	8.79	SNP_00965514	SNP_001633487	9.57	0.91	27.67	
59		LG5	DTM_S17	*qDTM6.I*	SNP_001630734	7.38	SNP_001042170	SNP_001633487	10.65	1.03	34.72	
60		LG5	DTM_S18	*qDTM6.I*	SNP_001867275	7.38	SNP_001633487	SNP_001867275	9.91	1.66	32.43	
61		LG5	VegP_GEI	*qVegP6.I*	SNP_004211395	3.25	SNP_004210056	SNP_004215482	18.57	1.18	43.54	

62		LG5	VegP_R17	*qVegP6.I*	SNP_001630734	7.38	SNP_001042170	SNP_001633487	21.81	1.28	53.52	
63		LG5	VegP_R18	*qVegP6.I*	SNP_001042170	8.79	SNP_00965514	SNP_001633487	10.62	0.73	32.36	
64		LG5	VegP_S17	*qVegP6.I*	SNP_000064101	3.25	SNP_00056583	SNP_000071367	16.58	1.53	45.60	
65		LG5	VegP_S18	*qVegP6.I*	SNP_000064101	3.25	SNP_00056583	SNP_000071367	16.10	0.98	43.16	
66		LG5	YLD_GEI	*qYLD6.I*	SNP_001630734	7.38	SNP_001042170	SNP_001633487	7.21	23.26	23.28	
67		LG5	YLD_R18	*qYLD6.I*	SNP_004590974	2.34	SNP_004590635	SNP_004590974	5.78	21.20	19.53	
68		LG5	YLD_S17	*qYLD6.I*	SNP_004210056	4.25	SNP_004210056	SNP_004215482	6.72	33.24	23.04	
69		LG5	YLD_S18	*qYLD6.I*	SNP_004590635	2.34	SNP_004210056	SNP_004590974	6.59	18.89	21.32	
70	LcChr6.II	LG5	RepP_R18	*qRepP6.II*	SNP_015003020	16.15	SNP_014455895	SNP_015056478	4.02	0.03	13.92	*Lcu.2RBY.6g003190*
71		LG5	YLD_R17	*qYLD6.II*	SNP_017355269	20.29	SNP_017270400	SNP_017817183	8.02	29.26	22.40	
72		LG5	DTM_R18	*qDTM6.II*	SNP_025462888	24.30	SNP_025460994	SNP_028554141	5.14	0.98	18.57	
73		LG5	RepP_S18	*qRepP6.II*	SNP_028765058	25.26	SNP_028765057	SNP_028765113	6.52	0.79	16.32	
74		LG5	RepP_R17	*qRepP6.II*	SNP_113648039	29.35	SNP_113647548	SNP_121924240	4.93	0.36	16.17	
75		LG5	RepP_GEI	*qRepP6.II*	SNP_106985116	30.80	SNP_106831978	SNP_107772025	7.41	0.33	18.17	
77	LcChr6.III	LG5	DTF_R18	*qDTF6.III*	SNP_419835768	132.94	SNP_419817975	SNP_419853726	4.21	0.29	13.98	
78		LG5	VegP_S17	*qVegP6.III*	SNP_419835768	132.94	SNP_419817975	SNP_419853726	4.35	0.65	17.76	
79		LG5	DTF_S18	*qDTF6.III*	SNP_000196688	133.82	SNP_000056583	SNP_000208076	6.23	0.52	21.44	
80		LG5	DTF_GEI	*qDTF6.III*	SNP_000298283	133.82	SNP_000298153	SNP_000306895	5.44	0.52	15.63	
81		LG5	VegP_GEI	*qVegP6.III*	SNP_000298283	133.82	SNP_000298153	SNP_000306895	4.14	0.54	10.48	
82		LG5	DTF_S17	*qDTF6.III*	SNP_000298283	133.82	SNP_000298153	SNP_000306895	5.91	0.81	15.62	
83	LcChr7.I	LG6	YLD_S18	*qYLD7.I*	SNP_012108547	28.79	SNP_012108306	SNP_012108611	4.02	16.31	15.17	
84	LcChr7.I	LG6	RepP_R18	*qRepP7.II*	SNP_000752614	37.92	SNP_000729892	SNP_000778241	4.51	0.07	15.92	
85	LcChr7.I	LG6	RepP_R18	*qRepP7.III*	SNP_489782902	54.00	SNP_489373840	SNP_490589791	5.54	0.14	6.71	
